# Detection and Antimicrobial Resistance of *Staphylococcus* Species from Chicken, Chicken Litter, and Humans in Addis Ababa, Ethiopia

**DOI:** 10.1155/2022/9084334

**Published:** 2022-05-25

**Authors:** Fufa Abunna, Biyansa Adugna, Takele Beyene Tufa, Dinka Ayana, Fanta D. Gutema, Hika Waktole, Fikru Regassa, Reta D. Abdi

**Affiliations:** ^1^Addis Ababa University, College of Veterinary Medicine and Agriculture, P. O. Box 34, Bishoftu, Oromia, Ethiopia; ^2^Samara University, College of Veterinary Medicine, P. O. Box 3015, Samara, Afar, Ethiopia; ^3^Department of Biomedical Sciences, College of Veterinary Medicine, Long Island University, Greenvale, NY 11548, USA

## Abstract

**Background:**

In veterinary medicine, three *Staphylococcus* species are of particular importance as primary causes of specific diseases; *S. aureus* (mastitis in ruminants, equine botryomycosis, and bumble foot in poultry), *S. hycus* (porcine exudative epidermitis), and *S. intermedius* (canine pyoderma). The disease conditions caused by *Staphylococcus* in poultry vary with site, route, and predisposing factors include wounds as a result of fighting/cannibalism, immunosuppression based on virus infection or parasite infestation, and bad husbandry conditions (overcrowding). The objectives of this study were to isolate and identify *Staphylococcus* spp from chicken and chicken litter and personnel at chicken farm and to determine the antimicrobial susceptibility profile of the isolates.

**Methods:**

A cross-sectional study was conducted on apparently healthy chickens, farm personnel, and chicken litter at poultry farms in Addis Ababa, Ethiopia. A total of 222 samples consisting of 101 cloacal swabs, 90 tracheal swabs, 17 pooled litter swabs, 7 nasal swabs, and 7 pooled hand and boot swabs were collected from six farms and examined for the presence of *Staphylococcus* species. Antimicrobial resistance against 10 antimicrobial agents was also conducted following recommended standard procedures.

**Results:**

Overall proportion of *Staphylococcus* was 64/222 (28.83%). Of the isolates, 40/64 (62.5%), 11/64 (17.2%), 3/64 (4.7%), and 10/64 (15.6%), were *S. aureus*, *S. hycus*, *S. intermedius*, and coagulase-negative staphylococci (CNS), respectively. Only one isolate of *S. aureus* was susceptible to all antimicrobials tested. Of the 10 antibiotics tested, the isolates demonstrated highest resistance against Penicillin G (96.9%) followed by Tetracycline (78.1%), and Amoxicillin and Erythromycin at the same level (65.6%). Conversely, the isolates were highly susceptible to Ciprofloxacin (95.3%) followed by Sulphamethoxazole-trimethoprim (85.9%). Out of 64 isolates, 61/64 (95.3%) were resistant to three or more antimicrobials tested. Of the isolates, 38/40 (95%) *S. aureus,* 10/11 (90.9%) *S. hycus*, 3/3 (100%) *S. intermedius*, and 10/10 (100%) CNS showed multidrug resistance.

**Conclusion:**

This study showed a considerable proportion of *Staphylococcus* spp in chicken litter and farm workers with a potential source of resistant *Staphylococcus* species, and more importantly multidrug resistance strains. Further studies on molecular characterization of the isolates will be essential to identify the resistant genes and establish epidemiological links in the transmission dynamics of resistant *Staphylococcus* species between poultry and humans.

## 1. Background

Staphylococci are facultative anaerobic, non-motile gram-positive cocci, which commonly form grape-like clusters [1, 2]. In humans and animals, many staphylococcal species are commensals on the skin and mucosal surfaces such as the upper respiratory tract, alimentary tract, and genitourinary tract [3]. In addition to their wide distribution, staphylococci can easily spread between different animal species, and between humans and animal species. The sources of infection are mainly contaminated foods, water and equipment, carrier, and clinically infected human and animals, and environment where the animals are crowded together. Various transmission routes have been described including direct, via the hands, contact with excretions or contact with nonliving objects (fomites), ingestion of contaminated food and water, aerosols, and via vectors [4, 5].

Among the staphylococci, several studies identified the most pathogenic LA-MRSA isolated from pig, veal, calf, and dairy farms and those persons with occupational contact to livestock, such as farmers, veterinarians or abattoir workers, and other persons with exposure to livestock [6]. Although poultry plays a major role in intensive animal husbandry, there are only limited studies available on the isolation of *Staphylococcus* from poultry and from food of poultry origin intended for human consumption. Staphylococcal infections in both animals and humans are commonly treated with antimicrobial agents, most often with *β*-lactam antibiotics. These antibiotics were initially highly effective against staphylococci, but *β*-lactamase-producing *Staphylococcus* isolates emerged in the mid-1940s, and their prevalence increased dramatically within a few years [7, 8]. Staphylococci organisms, especially *S. aureus* strains, are known to produce beta-lactamases and acquired resistance to mobile genetic elements, plasmids, and transposons which could possibly play a vital role in the emergence of multiple drug resistant [9]. In Ethiopia, few research studies have been reported in the country pertaining *Staphylococcus* isolation and identification from poultry and poultry farms. Therefore, the objectives of this study were to isolate and identify and to assess the antimicrobial susceptibility patterns of *Staphylococcus* spp from chicken litter and poultry farm personnel in Addis Ababa.

## 2. Materials and Methods

### 2.1. Study Area

The study was conducted in and around Addis Ababa. Addis Ababa is the capital city and administration centre for the Federal Democratic Republic of Ethiopia. Currently, there are 10 subcities “*Kifle Ketemas*” in Addis Ababa city administration delineated on the basis of geographical setup, population density, assets and service providers' distribution, and convenience for administration [10]. It is situated at the latitude of 9°3′ north and 38°43′ east longitudinally. It lies in the central high lands of Ethiopia at an altitude of 2400 m.a.s.l. It has an average rainfall of 1800 mm per annum. The annual average maximum and minimum temperatures were 26°C and 11°C, respectively, with an overall average of 18.7°C. Highest temperatures are reached in May. The main rainy season extends from June to September. It has a relative humidity varying from 70% to 80% during the rainy season and 40% to 50% during the dry season. Addis Ababa covers about 54,000 hectors of land with an average population of more than 3 million [11].

### 2.2. Study Design, Samples, and Sampling

A cross-sectional design was used to generate the desired data from a total of 222 samples, collected randomly from apparently healthy exotic chickens. The sample types were cloacal and tracheal swabs from both layers and broilers, pooled litter swabs from each farm and nasal and pooled hand and boot swabs from farm workers.

An informed verbal consent was obtained from farm owners and workers. Cloacal and tracheal swab samples were collected into a single screw capped test tube containing 10 ml of buffered peptone water as transporting media. Each sample was labelled with necessary information, including date of sampling, type of sample, source of sample (farm), and identification of the animal. Finally, the samples were properly packed in an ice box and transported to the microbiology laboratory at the College of Veterinary Medicine and Agriculture of Addis Ababa University, Bishoftu, for bacteriological analysis.

### 2.3. Isolation and Identification

The International Organization for Standardization, ISO 6888-3:2003, was employed for the isolation and identification of *Staphylococcus* species from swab samples. A loop full of the pre-enriched samples was streaked on a blood agar plates (BAP) enriched with 7% heparinised sheep blood and incubated at 37°C for 24–48 hours under aerobic conditions. The plates were examined for the presence of *Staphylococcus* colonies based on the morphological characteristics (creamy, greyish white, or yellow colonies) and haemolytic pattern. The presumed colonies were further subcultured on nutrient agar plates (NAP) and incubated at 37°C for 24–48 hours to obtain pure colonies. The pure colonies were transferred to nutrient agar slants and stored at 4°C for further biochemical and antimicrobial susceptibility tests. Identification of staphylococci and species assignment was done based on KOH test, Gram's staining, catalase test, oxidation and fermentation test, sugar fermentation (mannitol and maltose) tests, and coagulase test [12].

### 2.4. Antimicrobial Susceptibility Test

A sterile swab was dipped into the standardized suspension of bacteria, and excess fluid was expressed by pressing and rotating the swab firmly against the inside of the tube. The swab was streaked in three directions and continuously brushed over the Mueller Hinton agar, and inoculated plates were allowed to stand for 3–5 minutes. The discs were placed onto the agar surface using sterile forceps and gently pressed with the point of a sterile forceps to ensure complete contact with the agar surface, and the plates were incubated aerobically at 37°C for 16 hours–18 hours for all disc except for Vancomycin 24 hours. Inhibition zone diameters were measured, and values obtained from the Clinical laboratory standard institute [13].

### 2.5. Data Management and Analysis

The generated data were stored in Microsoft Excel and analysed using SPSS version 20 software. Descriptive statistics such as percentage, the proportion, and frequency distributions were applied to compute some of the data. Chi-square (*χ*^2^) was used to compare sample source and types.

## 3. Results

Of the 222 samples examined, 64 (28.8%), [95%CL:28.12–29.54] were positive for *Staphylococcus* species, from which 4/22 (18.2%), 21/59 (35.6%), 10/28 (35.7%), 14/62 (22.6%), 11/23 (47.8%), and 4/28 (14.3%) from AM, BD, EU, HM, SA, and TE farm, respectively, were positive for *Staphylococcus* species. There was statistically significantly difference observed in the isolation of *Staphylococcus* between different farms (*P*=0.046).

Staphylococci were isolated from 4/28 (15.69%) and 58/194 (31.71%) of samples from birds kept in cages and deep litter systems, respectively. The isolation rate of *Staphylococcus* in samples from layers (Bovans brown) was 10/57 (17.54%) while 45/134 (33.58%), 7/1 (741.18%), and 2/4 (14.28%) of the samples from broilers, litter, and farm workers, respectively, were positive for *Staphylococcus*. There was no significant association between isolation rate and housing system (*P*=0.103) or sample source (*P*=0.052) (Table 1).

Of the total, 64/222 (28.8%) *Staphylococcus* isolates, 33/101 (32.7%), 22/90 (24.44%), 7/17 (41.2%), and 2/7 (28.6%) were from the cloacal swab, tracheal swab, pooled litter swab, and nasal swab of farm attendants, respectively. No isolate was found from pooled hand and boot swab of farm attendants. There was no statistically significant difference in *Staphylococcus* isolation between different sample types (*P* value = 0.225) (Table 1).

Out of the 222 samples examined, *S. aureus*, S. hycus. *S. intermedius*, and CNS were detected in 40 (18.01%), 11 (4.95%), 3 (1.4%), and 10 (4.5%), respectively. Of the isolates, *S. aureus* 40/64 (62.5%) was the most dominant followed by *S. hycus* 11/64 (17.2%) and CNS 10/64 (15.6%), and lastly *S. intermedius* 3/64 (4.7%). *S. aureus* was isolated from all farms with the highest prevalence in farm BD, 19/59 (32.2%), and lowest in farm SA, 1/23 (3.4%). Other staphylococcal species, *S. hycus* was isolated only from three farms, BD 2/59 (3.4%), EU 3/28 (10.7%), and SA 6/23 (26.1%); *S. intermedius* and CNS were also isolated from three similar farms, EU, HM, and SA, with 1/28 (3.6%), 1/62 (1.6%), 1/23 (4.3%) *S. intermedius*, and 4/28 (14.3%), 3/62 (4.8%), and 3/23 (13%) CNS, respectively. There was statistical difference significantly in isolation and identification of *Staphylococcus* species between different farms except for *S. intermidius* (*P*=0.53) (Table 2).

From the two types of housing systems (cage and deep litter), 34/194 (18.6%) and 4/28 (14.3%) from deep litter and cage type housing system were positive for *S. aureus,* respectively, but S*. hycus*, 11/194 (5.7%), *S. intermedius*, 3/194 (1.6%), and CNS, 10/194 (5.2%) were only isolated from deep litter housing system, and there was no statistically significant difference in *Staphylococcus* species isolated and identified between housing system, since the *P* value in all species is (*P* value >0.05) (Table 2).

Among the S*taphylococcus* species, *S. aureus* was isolated from all sample sources, broiler (17.9%), layer (17.5%), litter (23.5%), and personnel (14.3%). Both *S. hycus* and CNS with the same result (6.7%) were isolated from broilers; S. *intermedius* was only isolated from broilers (2.2%). There was no statistically significant difference in *Staphylococcus* species isolated and identified between different sample sources (*P* value ≥0.05 in all species) (Table 2). All staphylococcal species were isolated from different sample types, but none of them were identified from the pooled hand and boot swabs of farm attendants. *S. aureus* was the highest (28.6%) in nasal swabs and lowest (12.9%) in cloacal swabs. *S. aureus* 4 (23.5%). *S. hycus* 2 (21.8%), and CNS 1 (5.9%) were isolated from litter, but *S. intermedius* was not. *S. intermedius* was only isolated from cloacal swab, 3/101 (3%). There was no statistically significant association observed in *Staphylococcus* species isolated from different sample types except CNS (*P*=0.05) (Table 2).

All isolates (64) of *Staphylococcus* were tested for susceptibility test to 10 antimicrobial discs. The comparative efficacies of antimicrobials used indicate that CIP and SXT were the most effective antibiotics with susceptibility percentage of 95.3% and 85.9%, respectively. Conversely, P and TE have shown the poorest efficacy (susceptibility) or high resistance against staphylococcal isolates with 96.9% and 78.1%, respectively, (Table 3).

All positive samples of *Staphylococcus* species, *S. aureus* (40), *S. hycus* (11), *S. intermedius* (3), and CNS (10) were tested for susceptibility. Of the isolates 38/40 (95%) *S. aureus*, 10/11 (90.9) *S. hycus*, 10/10 (100%) CNS, and 3/3 (100%) *S. intermedius* were resistance to three or more antimicrobials, while 1/40 (2.5%) *S. aureus* and 1/11 (9.1%) *S. hycus* showed monoresistance, and only 1/40 (2.5%) *S. aureus* was susceptible to all antimicrobials tested. *S. aureus* is highly resistant (95.0%) to P and (72.5%) to both VA and TE, and highly susceptible (97.5%), and (87.5%) to CIP and SXT, respectively. *S. hycus* showed greater resistance (100%) to P and highly resistance (81.8%) to TE; and greater susceptible (100%) to SXT and highly susceptible (90.9%) to CIP and slightly susceptible (63.6%) to both VA and NA. Similarly, *S. intermedius* showed greater resistance (100%) to P, AML, TE, and S, but greater susceptibility (100%) was seen in VA. Moreover, CNS showed greater resistance (100%) to P and greater susceptibility (100%) to CIP (Table 4).

Out of 64 *Staphylococcal* isolates, only one (1.56%) isolate *(S. aureus)* was susceptible to all tested antimicrobial agents, while two (3.13%) were found to be monodrug resistant. Multidrug resistance to three or more antimicrobials was observed in 61 (95.31%) of all isolates (Table 5).

## 4. Discussion

*Staphylococcus* spp. are significant bacteria in the aetiology of avian diseases, but little is known about the bacterial presence in the poultry environment such as in poultry litter and in the poultry house air as reported by Saleh et al. [14]. The modern poultry industry can produce market-ready broiler chickens in less than six weeks. This accomplishment has been achieved through genetic selection, improved feeding, and keen health management practices including the usage of antibiotics as therapeutic agents to treat bacterial diseases in intensive farming systems. Resistance to frequently used antibiotics has been observed in bacteria present in poultry since the introduction of these antimicrobial agents in poultry [15].

Out of a total of 222 samples, 64 (28.8%) were found to be positive for *Staphylococcus* species which is lower than the finding of Modestas et al. [16] who reported 71% *Staphylococcal* species from poultry products in Kaunas, Lithuania, but higher than 15.2% *Staphylococcal* species reported by Masdooq et al. [17] from pathogenic bacteria associated with respiratory diseases of poultry in Nigeria.

Of the total isolates, it was found that 62.5% of them were *S. aureus* which is higher than the previous work of [18] that reported the isolation of *S. aureus* (23.53%) from yolk sac infection (omphalitis) in Kombolcha poultry farm, Ethiopia, and [19] who reported 20.5% *S. aureus* from chicken in Jos, Nigeria. Moreover, the present finding revealed the prevalence of *S. aureus* (62.5%), *S. hycus* (17.2%) and, *S. intermedius* (4.7%) was nearly the same with 2%, and CNS (15.6%). In the previous study in Morocco, it was reported that *S. aureus* (40%), *Staphylococcus intermedius* (2%), and *Staphylococcus hyicus* (4%) were much lower than 54% which were (i.e., 40%, 4%, 2%, and 54%) the findings of [20] in Morocco. Other observation was also reported by [21] with a prevalence of 46% *S. aureus* from poultry products in Egypt which was of course lower than the present study value.

Of the samples collected from broiler, layer, litter, and farm attendants, 45 (33.58%), 10 (17.54%), 7 (41.18%), and 2 (14.29%), respectively, were found to be positive for *Staphylococcus species*. Similarly, Saleh et al. [14] reported that the prevalence of *Staphylococcus* in broiler was 32% which is comparable with our finding (33.58%) in broilers. Moreover, they have detected 35% *Staphylococcus species* in layers which is much higher than the present result (17.54%). In our study, the *S. aureus* isolated from broiler and layers was 17.9% and 17.5%, respectively, which is much lower than the finding of [22] who reported 50.89% *S. aureus* from broilers in Iraq. Similarly, the present finding of *S. aureus* from litter (23.5%) was lower than the finding of [23] who reported 33.3% in Khartoum state. In the present study, the detection of *S. aureus* from nasal swabs (28.8%) is higher than the finding of [24] who reported 3.61% from nasal swabs of poultry farm workers in Iran; however, the detection of the isolate from tracheal swab (23.3%) was much lower than the finding of [25] that reported 85% of *S. aureus* from tracheal swab. The variation in detection of the isolates may emanate from several factors; the variation in the diagnostic capability of the laboratories may vary from country to country, geographical variations, variation in breeds, management practices, etc.

In this study, 95.0% *S*. *aureus* isolates were found to be resistant to Penicillin G. A previous study also revealed a similar finding where 96.7% *S. aureus* were found to be resistant to Penicillin G [26] but slightly lower than 100% resistance reported by Otalu et al. [27]. In this study, 100% resistance towards Penicillin G is recorded by *S. hycus*, and CNS showed the highest resistance. This finding is in consistent with that of [20] who reported similar results from milk and whey in Morocco. Thus, the results indicate that the majority of antimicrobial resistance in *S. aureus* and CNS isolates could be due to the production of *β*-lactamases and may carry the *mecA* chromosomal gene responsible for the production of the altered penicillin binding protein PBP-2a as suggested by [15].

Of the total isolates (64) subjected to the antimicrobial susceptibility test, 61/64 (95.3%) have developed multidrug resistance (resistant to three and more than three antimicrobials). High prevalence of multidrug resistance among isolates in the present study clearly indicated the excessive or inappropriate use of antibiotics. This may be connected to the fact that antimicrobial use in commercial poultry settings is more likely to be regulated than in small holder farms. In addition, veterinarians and poultry farmers generally use these antimicrobials as prophylaxes, growth promoters, or inaccurate dosages given to sick flocks by unqualified personnel may likely result a high level of resistance that was reported by [25] done on antimicrobial resistant *Staphylococcus* from chickens in Maiduguri, Nigeria, and it is in agreement with present finding of multi-drug resistance.

## 5. Conclusion

The results of the current study showed that *Staphylococcus* is one of the organisms that are widely distributed in poultry farms in the study area. In this study, a total of 222 samples were examined and processed, and 64 (28.83%) *Staphylococci* were isolated and of which *S. aureus* was the most dominant one, 40 (62.5%), even though little or no comparative report in Ethiopia at all. The antimicrobial susceptibility test of the *Staphylococcus* isolates in this study showed that 61/64 (95.31%) isolates, almost all were multidrug resistance (three and more than three antimicrobials tested), and this makes an alarming cause for further studies. Therefore, further studies should be conducted on a large scale to find the association between source of infection and prevalence to find out the possible source of contaminati*on* with *Staphylococcus* species, and antimicrobial susceptibility test should be carried out at regular intervals to determine the development of resistance against the most commonly applied antibiotics.

## Figures and Tables

**Table 1:** Distribution of *Staphylococcus* isolates in different farms, housing systems, sample source, and sample types.

		Positive	Total	Prevalence (%)	95%CI	*χ* ^2^	*P*
Farm name	M	4	22	18.18	16.40, 19.96	11.92	0.046
	D	21	59	35.59	34.07, 37.12		
	U	10	28	35.71	33.50, 37.93		
	M	14	62	22.58	21.40, 23.76		
	A	11	23	47.83	45.00, 50.65		
	E	4	28	14.29	12.89, 15.69		

Housing system	C	4	28	14.29	12.89, 15.69	3.30	0.07
	L	60	194	30.93	30.15, 31.71		

Sample source	B	45	134	33.58	32.60, 34.56	7.72	0.052
	L^*∗*^	7	17	41.18	38.13, 4.23		
	Ly	10	57	17.54	16.46, 18.63		
	P	2	14	14.29	12.31, 16.27		

Sample type	C	33	101	32.67	31.56, 33.79	5.67	0.225
	HB	0	7	0.00	—		
	L^*∗*^	7	17	41.18	38.13, 44.23		
	N	2	7	28.57	24.61, 32.53		
	T	22	90	24.44	23.42, 25.47		
Total		64	222	28.83	28.12, 29.54		

Keys: AM = Amelewerk farm enterprise; BD = Bayissa Damessa farm; EU = Europe farm enterprise; HM = Haile Michael farm; SA = Senait and Abdella and their friends; TE = Tesfaye farm enterprise; C = cage type housing system; L = litter type housing system; P = personnel, Ly = layer, B = broiler, Cl = cloacal swab; L^*∗*^ = litter swab, T = tracheal swab; N = nasal swab; HB = hand and boot swab.

**Table 2:** The proportional distribution of *Staphylococcus* species isolated from different farms, housing systems, sample sources, and different sample types.

		*Staphylococcus* species, *n* (%)			
		CNS	SA	SH	SI
Farm name	AM (*n* = 22)	0 (0.0)	4 (18.2)	0 (0.0)	0 (0.0)
	BD (*n* = 59)	0 (0.0)	19 (32.2)	2 (3.4)	0 (0.00
	EU (*n* = 28)	4 (14.3)	2 (7.1)	3 (10.7)	1 (3.6)
	HM (*n* = 62)	3 (4.8)	10 (16.1)	0 (0.0)	1 (1.6)
	SA (*n* = 23)	3 (13)	1 (3.4)	6 (26.1)	1 (4.3)
	TE (*n* = 28)	0 (0.0)	4 (14.3)	0 (0.0)	0 (0.0)

*χ* ^2^ (*P* − *v*)		15.28 (0.01)	13.60 (0.02)	29.93 (0.00)	4.12 (0.53)

Housing system	C (*n* = 28)	0 (0.0)	4 (14.3)	0 (0.0)	0 (0.00
	L (*n* = 194)	10 (5.2)	36 (18.6)	11 (5.7)	3 (1.6)

*χ* ^2^ (*P* − *v*)		2.30 (0.32)	0.32 (0.88)	2.55 (0.29)	0.67 (0.72)

Sample source	B (*n* = 134)	9 (6.7)	24 (17.9)	9 (6.7)	3 (2.2)
	L (*n* = 17)	1 (5.9)	4 (23.5)	2 (11.8)	0 (0.0)
	Ly (*n* = 57)	0 (0.0)	10 (17.5)	0 (0.00	0 (0.0)
	P (*n* = 14)	0 (0.0)	2 (14.3)	0 (0.0)	0 (0.0)

*χ* ^2^ (*P* − *v*)		4.95 (0.18)	0.50 (0.92)	6.26 (0.10)	2.00 (0.57)

Sample type	C (*n* = 101)	9 (8.9)	13 (12.9)	8 (7.9)	3 (3)
	HB (*n* = 7)	0 (0.0)	0 (0.0)	0 (0.0)	0 (0.0)
	L (*n* = 17)	1 (5.9)	4 (23.5)	2 (11.8)	0 (0.0)
	N (*n* = 7)	0 (0.0)	2 (28.6)	0 (0.0)	0 (0.0)
	T (*n* = 90)	0 (0.0)	21 (23.3)	1 (1.1)	0 (0.0)

*χ* ^2^ (*P* − *v*)		9.54 (0.05)	5.95 (0.20)	7.11 (0.13)	3.64 (0.46)

Total	*n* = 64	10 (15.6)	40 (62.5)	11 (17.2)	3 (4.7)

Key: CNS = coagulase negative *Staphylococcus*; SA = *S*. *aureus*; SH = *S. hycus*; SI = *S. intermedius*; *χ*^2^ = chi-square, *P* = *P* value, CI = confidence interval, and C = cage type housing system; L = litter-type housing system; P = personnel, Ly = layer, B = broiler, and Cl = loacal swab; L = litter swab and T = tracheal swab; N = nasal swab; HB = hand and boot swab.

**Table 3:** Antimicrobial susceptibility profile of staphylococcal isolates (*n* = 64).

S(*n*)	Antimicrobials	Number of tested	Susceptible [*N* (%)]	Intermediate [*n* (%)]	Resistance [*N* (%)]
1	P	64	2 (3.1)	0 (0.0)	62 (96.9)
2	VA	64	26 (40.6)	0 (0.0)	38 (59.4)
3	CIP	64	61 (95.3)	0 (0.0)	3 (4.7)
4	SXT	64	55 (85.90	0 (0.0)	9 (14.1)
5	AML	64	22 (34.4)	0 (0.0)	42 (65.6)
6	F	64	19 (29.70	27 (42.2)	18 (28.1)
7	E	64	6 (9.4)	16 (25.0)	42 (65.6)
8	TE	64	7 (10.9)	7 (10.9)	50 (78.1)
9	S	64	24 937.5)	16 (25.0)	24 (37.5)
10	NA	64	48 (75.0)	5 (7.80)	11 (17.2)
Total	10	640	270 (42.2)	71 (11.1)	299 (46.7)

Key: P = penicillin, AML = amoxicillin, F = cefoxitin, CIP = ciprofloxacin, NA = naldixic acid, S = streptomycin, SXT = sulfamethoxazole trimethoprim, VA = vancomycin, TE = tetracycline, and E = erythromycin.

**Table 4:** Antimicrobial susceptibility patterns of *Staphylococcus* species.

Antimicrobials tested		*Staphylococcus* species [*n* (%)]					
		CNS	SA	SH	SI	*χ* ^2^	*P*
P	R (*n* = 62)	10 (100)	38 (95.0)	11 (100)	3 (100)	1.239	0.744
	S (*n* = 2)	0 (0.0)	2 (5.0)	0 (0.0)	0 (0.0)		

VA	R (*n* = 38)	5 (50.0)	29 (72.5)	4 (36.4)	0 (0.0)	10.02	0.018
	S (*n* = 26)	5 (50.0)	11 (27.5)	7 (63.6)	3 (100)		

CIP	R (*n* = 3)	0 (0.0)	1 (2.5)	1 (9.1)	1 (33.3)	6.908	0.075
	S (*n* = 62)	10 (100)	39 (97.5)	10 (90.9)	2 (66.7)		

SXT	R (*n* = 9)	2 (20.0)	5 (12.5)	0 (0.0)	2 (66.7)	9.042	0.029
	S (*n* = 55)	8 (80.0)	35 (87.5)	11 (100)	1 (33.3)		

AML	R (*n* = 42)	8 (80.0)	26 (65.0)	5 (45.5)	3 (100)	4.478	0.214
	S (*n* = 22)	2 (20.0)	14 (35.0)	6 (54.5)	0 (0.0)		

F	I (*n* = 27)	1 (10.0)	21 (52.5)	5 (45.5)	0 (0.0)	16.032	0.014
	R (*n* = 18)	7 (70.0)	6 (15.0)	4 (36.4)	1 (33.3)		
	S (*n* = 19)	2 (20.0)	13 (32.5)	2 (18.2)	2 (66.7)		

E	I (*n* = 16)	1 (10.0)	0 (0.0)	0 (0.0)	1 (33.3)	20.119	0.003
	R (*n* = 42)	9 (90.0)	25 (62.5)	7 (63.6)	1 (33.3)		
	S (*n* = 6)	0 (0.0)	1 (2.5)	4 (36.4)	1 (33.3)		

TE	I (*n* = 7)	0 (0.0)	7 (17.5)	0 (0.0)	0 (0.0)	5.642	0.465
	R (*n* = 50)	9 (90.0)	29 (72.5)	9 (81.8)	3 (100)		
	S (*n* = 7)	1 (10.0)	4 (10.0)	2 (18.2)	0 (0.0)		

S	I (*n* = 16)	2 (20.0)	11 (27.5)	3 (27.3)	0 (0.0)	7.27	0.297
	R (*n* = 24)	4 (40.0)	15 (37.5)	2 (18.2)	3 (100)		
	S (*n* = 24)	4 (40.0)	14 (35.0)	6 (54.5)	0 (0.0)		

NA	I (*n* = 5)	0 (0.0)	4 (10.0)	1 (9.1)	0 (0.0)	4.931	0.553
	R (*n* = 11)	3 (30.0)	4 (10.0)	3 (27.3)	1 (33.3)		
	S (*n* = 48)	7 (70.0)	32 (80.0)	7 (63.6)	2 (66.7)		
Total		10 (15.6)	40 (62.5)	11 (17.2)	3 (4.7)		

*Note.* I = intermediate, R = resistant, and S = susceptible.

**Table 5:** Number and percentages of antimicrobial resistance patterns of *Staphylococcus* species.

No. of antimicrobials	Antimicrobial resistance pattern	Number (%)	Species
One	NA	1 (1.56)	SH
	P	1 (1.56)	SA
Three	P, E, S(1); P, AML, TE (1); P, E, TE(1); P, VA, TE (2); P, F, E (1); P, VA, E (1)	7 (10.94)	SA
	P, AML, TE(1) P, E, TE(1)	2 (3.13)	SH
Four	P, AML, E, TE (2); P, CIP, AML, NA (1) P, E, TE, NA (1)	4 (6.25)	SH
	P, AML, F, TE (1); P, AML, E, S (1)	2 (3.13)	CNS
	P, AML, F, TE (1); P, VA, AML, TE (2); P, VA, AML, E (5); P, VA, E, S (1); P, VA, TE, S (1) P, E, TE, S (1)	11 (17.19)	SA
Five	P, CIP, AML, TE, E (1)	1 (1.56)	SI
	P, VA, F, E, TE (2);	2 (3.13)	SH
	P, AML, F, E, TE (1); P, VA, AML, E, TE(1); P, VA, E, TE, NA (1)	3 (4.69)	CNS
	P, AML, F, E, TE (1); P, VA, AML, E, TE(4); P, VA, SXT, AML, TE (1); P, VA, AML, TE, S (2); P, VA, TE, S, NA (1); P, VA, E, TE, S (1); P, AML, F, TE, S (1)	11 (17.19)	SA
Six	P, SXT, AML, E, TE, S (1);	1 (1.56)	SI
	P, VA, F, E, TE, S (1);	1 (1.56)	SH
	P, VA, AML, F, E, TE (1); P, AML, F, E, TE, NA (1)	2 (3.13)	CNS
	P, SXT, AML, E, TE, S (1); P, VA, AML, F, E, TE (1); P, VA, SXT, E, TE, S (1); P, VA, SXY, AML, TE, S (1); P, VA, AML, E, TE, S (2)	6 (9.380)	SA
Seven	P, SXT, AML, F, TE, S, NA (1);	1 (1.56)	SI
	P, VA, AML, F, TE, S, NA (1);	1 (1.56)	SH
	P, SXT, AML, F, E, TE, S (1) P, VA, AML, F, E, TE, S (1);	2 (3.13)	CNS
	P, VA, AML, F, E, TE, S (1); P, VA, SXT, AML, E, TE, NA (1); P, VA, CIP, AML, E, TE, NA (1)	3 (4.69)	SA
Eight	P, VA, SXT, F, E, TE, S, NA(1)	1 (1.56)	CNS
Total	Monoresistance (2) Multiresistance (61)	63 (98.44)	SA (39)
			SH (11)
			SI (3)
			CNS (10)
MDR	61	61 (95.31)	SA (38)
			SH (10)
			SI (3)
			CNS (10)

Key: MDR = multidrug resistance, SA = *S*. *aureus*, SH = *S. hycus*, and SI = *S. intermedius*.

## Data Availability

The data can be obtained from the corresponding author.
